# Trends in Prevalence and Severity of Pre/Perinatal Cerebral Palsy Among Children Born Preterm From 2004 to 2010: A SCPE Collaboration Study

**DOI:** 10.3389/fneur.2021.624884

**Published:** 2021-05-20

**Authors:** Catherine Arnaud, Virginie Ehlinger, Malika Delobel-Ayoub, Dana Klapouszczak, Oliver Perra, Owen Hensey, David Neubauer, Katalin Hollódy, Daniel Virella, Gija Rackauskaite, Andra Greitane, Kate Himmelmann, Els Ortibus, Ivana Dakovic, Guro L. Andersen, Antigone Papavasiliou, Elodie Sellier, Mary Jane Platt, Inge Krägeloh-Mann

**Affiliations:** ^1^UMR 1027 SPHERE Team, Inserm, Toulouse 3 Paul Sabatier University, Toulouse, France; ^2^Childhood Disability Registry in Haute-Garonne, University Hospital of Toulouse, Toulouse, France; ^3^Clinical Epidemiology Unit, University Hospital of Toulouse, Toulouse, France; ^4^School of Nursing & Midwifery and Centre for Evidence and Social Innovation, Queen's University Belfast, Belfast, United Kingdom; ^5^Central Remedial Clinic, Dublin, Ireland; ^6^Department of Child, Adolescent & Developmental Neurology, University Children's Hospital Ljubljana, Ljubljana, Slovenia; ^7^Department of Paediatrics, Medical School, University of Pécs, Pécs, Hungary; ^8^Neonatology Intensive Care Unit/Research Center, Central Lisbon Hospital, Lisbon, Portugal; ^9^Child and Adolescent Medicine, Aarhus University Hospital, Aarhus, Denmark; ^10^Association Rehabilitation Center, Riga, Latvia; ^11^Department of Pediatrics, Clinical Sciences, Sahlgrenska Academy, University of Gothenburg, Gothenburg, Sweden; ^12^Department of Development and Regeneration, KU Leuven, Leuven, Belgium; ^13^Children's Hospital Zagreb, Medical School, University of Zagreb, Zagreb, Croatia; ^14^The Norwegian University of Science and Technology, NTNU, Trondheim, Norway; ^15^The Cerebral Palsy Register of Norway, Vestfold Hospital Trust, Tønsberg, Norway; ^16^Department of Paediatric Neurology, IASO Children's Hospital, Athens, Greece; ^17^Grenoble Alpes University, CNRS, Grenoble INP, CHU Grenoble Alpes, TIMC-IMAG, Grenoble, France; ^18^Registre des Handicaps de l'Enfant et Observatoire Périnatal, Grenoble, France; ^19^Norwich Medical School, University of East Anglia, Norwich, United Kingdom; ^20^Department of Paediatric Neurology and Developmental Medicine, University Children's Hospital, Eberhard Karls Universität Tübingen, Tübingen, Germany

**Keywords:** cerebral palsy, preterm birth, prevalence, severity, brain imaging

## Abstract

**Aim:** To report on prevalence of cerebral palsy (CP), severity rates, and types of brain lesions in children born preterm 2004 to 2010 by gestational age groups.

**Methods:** Data from 12 population-based registries of the Surveillance of Cerebral Palsy in Europe network were used. Children with CP were eligible if they were born preterm (<37 weeks of gestational age) between 2004 and 2010, and were at least 4 years at time of registration. Severity was assessed using the impairment index. The findings of postnatal brain imaging were classified according to the predominant pathogenic pattern. Prevalences were estimated per 1,000 live births with exact 95% confidence intervals within each stratum of gestational age: ≤27, 28–31, 32–36 weeks. Time trends of both overall prevalence and prevalence of severe CP were investigated using multilevel negative binomial regression models.

**Results:** The sample comprised 2,273 children. 25.8% were born from multiple pregnancies. About 2-thirds had a bilateral spastic CP. 43.5% of children born ≤27 weeks had a high impairment index compared to 37.0 and 38.5% in the two other groups. Overall prevalence significantly decreased (incidence rate ratio per year: 0.96 [0.92–1.00[) in children born 32–36 weeks. We showed a decrease until 2009 for children born 28–31 weeks but an increase in 2010 again, and a steady prevalence (incidence rate ratio per year = 0.97 [0.92–1.02] for those born ≤27 weeks. The prevalence of the most severely affected children with CP revealed a similar but not significant trend to the overall prevalence in the corresponding GA groups. Predominant white matter injuries were more frequent in children born <32 weeks: 81.5% (≤27 weeks) and 86.4% (28–31 weeks), compared to 63.6% for children born 32–36 weeks.

**Conclusion:** Prevalence of CP in preterm born children continues to decrease in Europe excepting the extremely immature children, with the most severely affected children showing a similar trend.

## Introduction

Cerebral palsy (CP) is a group of disorders of various etiologies that have movement and posture disturbances in common. The clinical picture includes a wide range of deficits of varying severity that have a significant impact on daily lives, and that persist through the lifespan (1). CP occurs as a result of abnormal brain development or damage to the developing brain. The increasing use of early magnetic resonance imaging (MRI) allows a better comprehension of the underlying pathophysiological mechanisms (2, 3). Children born preterm (PT) i.e. before 37 completed weeks of gestation are at increased risk of developing CP, and the risk increases with higher immaturity at delivery (4, 5). Meta-analyses of studies that addressed the relationship between length of gestation and prevalence of CP for the generations born 1985 to 2002/2004 showed that children born at 32–36, 28–31, and ≤27 weeks were, respectively, 5–6, 32–54, and 60–130 times more likely to have CP than full-term children (6, 7). These findings are in line with a considerable number of studies demonstrating that children born extremely preterm (EPT, ≤27 weeks) presented neurodevelopmental disabilities with higher incidence and severity than term-born children (8–14).

Over decades it has been commented on CP prevalence is unchanging. Only slowly have reports about decreasing prevalence come to recognition. First this was reported in children with low birthweight (LBW, <2,500 g) in Sweden and Germany (15). In children born in the late 80ies and after, a decrease in prevalence was reported among those with moderately LBW (MLBW, 1,500–2,499 g) in Europe (16, 17) and Australia (18). Also in children with very low birthweight (VLBW, 1,000–1,499 g), prevalence decreased starting in the 80ies in Europe (16, 19) and about one decade later in Australia (18, 20). This decrease in prevalence then also led to a decrease concerning the overall prevalence of CP (16, 18, 20–25). However, it remains unclear whether this downward trend benefits children with extremely low birthweight (ELBW, <1,000 g).

Among children born preterm, there is also limited knowledge on severity of CP across GA groups (6). In studies examining long-term trends, there is some evidence that the prevalence of severe forms has decreased over time in children born PT or with a LBW with an increasing rate of ambulant children and fewer concomitant disabilities reported (16, 18). However, in children who were born EPT or with ELBW, the severity rates seemed to remain steady over the studied periods (16), and declining trends were only observed in associated intellectual impairment (18).

Most children with CP who were born very preterm (VPT) have neuroimaging evidence of predominant white matter injury (26). Standardizing terminology and classification allows to heighten the knowledge of the nature of brain lesions at population's level. A very first analysis using the MRI Classification System (MRICS) developed and validated by the Surveillance of Cerebral Palsy in Europe (SCPE) collaboration (27) showed a very clear lesional pattern in children born VPT with 80% having predominant white matter lesions (such as periventricular leucomalacia or sequelae of hemorrhage). Data also suggested that this proportion increases with decreasing GA at birth (28).

Harmonized and reliable population-based data on children with CP from population-based registries from across Europe are routinely collected as part of the SCPE network. These have the advantage of providing a consistent and clear definition of the condition, a detailed clinical description with severity standardly described by functional scores, and a classification for MRI findings. The aim of this study was to use 2004 to 2010 birth cohorts to report on prevalence of CP, severity rates, and types of brain lesions in children born preterm overall and stratified by GA groups.

## Materials and Methods

### Source of Data

We used data from population-based registries participating in the SCPE network, covering either a part or their whole country (https://eu-rd-platform.jrc.ec.europa.eu/scpe). These registries provide yearly pseudonymized data on children with CP to the SCPE central database. Population data (live births and neonatal mortality the same year in the same catchment area) are available from the census or any official data population source. The SCPE central database is stored and managed by the European Commission Joint Research Center, Health in Society Unit, Directorate Health, Consumers and Reference Materials, Ispra (Italy). Definitions, classifications, data collection and harmonization methods have been reported elsewhere (29–32).

### Population Studied

Children with CP were eligible for this study if they were born preterm (<37 weeks GA) between 2004 and 2010 (2010 was the most recent year validated at European level), had a pre/perinatal CP according to SCPE definition (30) (children with CP of post neonatal origin, i.e., a recognized brain damaging event that is unrelated to factors in the ante-, peri-, or neonatal periods, were excluded), and were at least 4 years old at time of registration. Children included in the analyses were born from mothers living in the catchment areas at the time of birth. Registries were eligible if the number of live births <37 weeks' gestation stratified by GA groups (EPT, ≤27 weeks; VPT, 28–31 weeks; moderately preterm, MPT, 32–36 weeks) were available for the geographical areas covered. Centers with <5,000 average annual livebirths between 2004 and 2010 were not considered. Within each registry, birth years with more than 25% missing data on GA on children with CP were excluded.

### Study Variables

The following characteristics were collected. Perinatal data included mother's age at birth (in years), multiple birth (singletons vs. twins or higher multiple), cesarean delivery (yes/no), GA in completed weeks, sex. CP subtype was classified according to the predominant neurological clinical findings into spastic CP (bilateral or unilateral), dyskinetic CP, and ataxic CP or unknown (https://eu-rd-platform.jrc.ec.europa.eu/scpe/data-collection/cp-subtypes_en). The severity of motor impairment was assessed using the Gross Motor Function Classification System (GMFCS) (33). GMFCS levels were grouped into 3 categories, I-II (ability to walk), III (walk with aids), and IV-V (wheelchair). The following co-morbidities were also considered: moderate to severe intellectual disability (based either on a formal intelligence quotient (IQ) testing or on a clinical estimate corresponding to an IQ < 50), severe visual impairment (blind or no useful vision in both eyes), severe hearing impairment (bilateral hearing loss >70 dB), active epilepsy (history of unprovoked seizures, still on treatment at age of registration). Severity was characterized using several options. First, we reported separately the percentage of children affected by moderate to severe intellectual disability, severe visual impairment, or active epilepsy. Second, children were classified according to their impairment index (34) into highly impaired (GMFCS IV-V and/or IQ < 50, whatever existence and severity of other impairments), lowly impaired (GMFCS I-II and IQ > 70, without any visual or hearing impairment, and no epilepsy), moderately impaired corresponding to all other conditions. We also recorded data on brain MRI performed post-neonatally and classified the findings according to the MRICS based on the predominant pathogenic pattern into maldevelopments, predominant white matter injury, predominant gray matter injury, miscellaneous, and normal (27).

### Analysis

All the analyses were stratified according to GA groups: ≤27, 28–31, 32–36 weeks. Maternal and perinatal characteristics, CP subtype, and impairments were described using counts and percentages calculated on non-missing data. They were compared between GA groups using chi-square tests. Prevalences were estimated per 1,000 live births and presented with exact 95% confidence intervals (CI).

We examined the overall prevalence time trend of CP and severe CP using negative binomial regression models considering the number of children with CP by registry and by year as the dependent variable and the number of live births per year in each registry as offset term. The model incorporated a random intercept that was specific to each registry, allowing the average level of prevalence over the period to vary between registries. The linearity of the average trend was tested by comparing a model with year of birth as a continuous variable and a more general model where the time trend was smoothed using restricted cubic splines with 3 knots (thus allowing a change in slope every 3-year period) (35). Between registries random variations around the average time trend were then tested by adding a random slope (random slope allows each registry time trend to deviate from the overall time trend) to the regression model. Three covariance matrixes were examined: unstructured, exchangeable, and independent which made different assumptions about variances and covariances of the random effects. We concluded that the average time trend differed between registries if one of these models better fitted the data than the random intercept model. All comparisons were based on the Akaike information criterion (AIC) values. A sensitivity analysis excluding observations (i.e., birth cohort in a given registry) with more than 10% of missing GA was carried out.

Analyses were performed using STATA software (version 14.0 Stata Corp., College Station TX, USA).

## Results

Among the 18 registries that contributed to the SCPE database for birth years 2004–2010, 12 had adequate size and denominators by GA available. After exclusion of birth years (at registry's level, median of 4.4% of missing GA by birth year and by registry), and cases (at child's level) with missing GA, the study sample comprised 2,273 individuals with pre/perinatal CP born <37 weeks (Table 1). In total, 22.0, 38.2, and 39.9% were born EPT, VPT, and MPT, respectively. Their characteristics are presented in Table 2 according to GA groups. In total, 25.8% were born from multiple pregnancies. About 2-thirds had a bilateral spastic CP. The proportion of those with unilateral spastic CP slightly increased from 22.1% in children born EPT to 31.4% in those born MPT. The distribution of GMFCS categories significantly differed between children born EPT and MPT. The proportion of children unable to walk even with aids (GMFCS IV-V) increased with decreasing GA at birth. Associated impairments were most frequent in children born EPT: 32.1% with IQ < 50, 12.3% with severe visual impairment, as compared to other GA groups, except for active epilepsy, most frequent in children born MPT. Whatever GA group, the proportion of children with associated impairments increased with increasing loss of gross motor function (Supplementary Table 1). The distribution of impairment index significantly differed across GA groups. Overall, 43.5% born EPT had a severe phenotype (high impairment index) compared to 37.0 and 38.5% in the two other groups.

**Table 1:** Number of children with cerebral palsy born preterm (<37 weeks gestation) between 2004 and 2010 and corresponding live births, and total annual live births, by registry.

**Location of the registry**	**Birth-years available**	**Total annual live births^†^**	***n* live births <37 WG**	***n* children with CP <37 WG**
C05, Northern Ireland, UK	2004–2010	24,032	11,876	122
C06, Western Sweden	2004–2010	25,122	9,822	127
C07, Counties of Dublin, Kildare and Wicklow, Ireland	2004–2008; 2010	25,184	8,398	136
C12, Denmark (Faroe Island and Greenland excluded)	2004–2007	64,714	17,493	188
C15, Norway	2004–2010	59,831	28,006	316
C19, Slovenia	2004–2009	19,675	8,557	100
C21, Portugal	2004–2006; 2008–2010	105,014	48,611	366
C22, Riga, Latvia	2004–2008	7,349	1,816	19
C23, South West Hungary	2004–2010	8,562	6,127	46
C27, Belgium	2004–2010	75,586	42,072	353
C28, Croatia	2004–2010	36,387	14,162	252
C31, Attica (Athens Metropolitan area), Greece	2004–2010	41,118	27,138	248
Total			224,078	2,273

*WG, weeks gestation*.

† *Average of the total annual number of live births over the study period*.

**Table 2:** Characteristics of children with cerebral palsy born 2004–2010, by gestational age groups, *n* = 2,273.

	**EPT ≤27 WG *n* = 499**	**VPT 28–31 WG *n* = 868**	**MPT 32–36 WG *n* = 906**	***p*-value (chi-square)**
Maternal age at birth, in years, *n* (%)				0.416
<20 20–29 30–34 35–39 >39 Missing, *n*	21 (5.6) 158 (42.1) 107 (28.5) 70 (18.7) 19 (5.1) 124	28 (4.1) 288 (41.8) 213 (30.9) 122 (17.7) 38 (5.5) 179	23 (3.2) 295 (40.7) 243 (33.6) 135 (18.6) 28 (3.9) 182	
Multiple birth, *n* (%) Missing, *n*	143 (29.3) 11	240 (28.1) 13	192 (21.7) 23	0.001
Cesarean delivery, *n* (%) Missing, *n*	231 (53.1) 64	489 (62.9) 91	488 (59.3) 83	0.004
Sex, male, *n* (%)	307 (61.5)	527 (60.7)	518 (57.2)	0.182
CP-subtype, *n* (%)				<0.001
Bilateral spastic Unilateral spastic Dyskinetic Other forms/unable to classify Missing, *n*	326 (65.5) 110 (22.1) 23 (4.6) 39 (7.8) 1	617 (71.1) 204 (23.5) 27 (3.1) 20 (2.3) 0	533 (58.9) 284 (31.4) 48 (5.3) 40 (4.4) 1	
GMFCS, *n* (%)				0.054
I-II, able to walk III, walk with aids IV-V, wheelchair Missing, *n*	264 (55.2) 67 (14.0) 147 (30.8) 21	480 (56.5) 118 (13.9) 251 (29.6) 19	546 (62.3) 96 (10.9) 235 (26.8) 29	
Moderate to severe intellectual disability (IQ <50), *n* (%) Missing, *n*	139 (32.1) 66	171 (22.3) 102	228 (28.5) 105	0.001
Severe visual impairment, *n* (%) Missing, *n*	53 (12.3) 68	61 (7.8) 88	65 (8.1) 103	0.019
Severe hearing impairment, *n* (%) Missing, *n*	24 (5.7) 78	26 (3.6) 147	27 (3.6) 160	0.162
Active epilepsy, *n* (%) Missing, *n*	110 (25.6) 70	148 (18.8) 80	232 (28.2) 83	<0.001
Impairment index^a^, *n* (%)				0.003
High Moderate Low Missing, *n*	202 (43.5) 171 (38.3) 73 (16.4) 53	294 (37.0) 313 (39.4) 188 (23.6) 73	316 (38.5) 351 (42.8) 154 (18.8) 85	

*EPT, extremely preterm; VPT, very preterm; MPT, moderately preterm; CP, Cerebral Palsy; WG, weeks gestation; GMFCS, Gross Function Classification System; IQ, Intellectual quotient*.

a *Impairment index: high (GMFCS IV-V and/or IQ <50 intellectual impairment, with or without one or more of the following impairments: severe visual impairment, severe hearing impairment, and active epilepsy; low (GMFCS I-II and IQ>70, without visual or hearing impairment, no epilepsy); moderate impairment (all other conditions)*.

**Figure 1:**
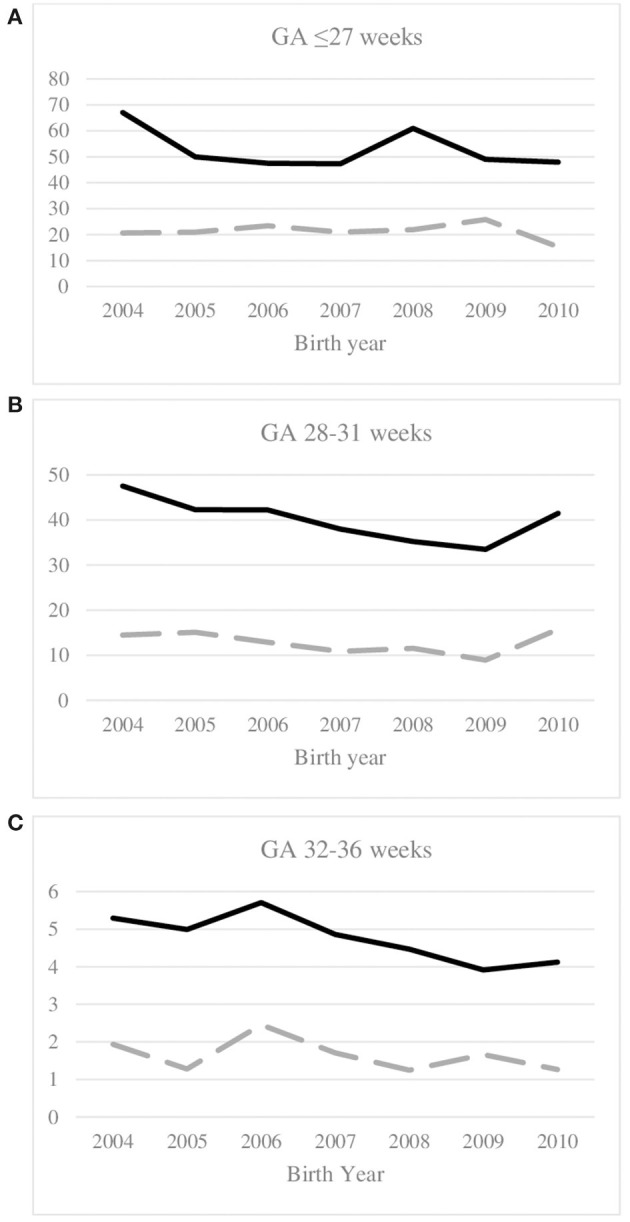
Time trends of overall prevalence of CP (full line) and prevalence of serve CP (dotted line) per 1,000 live births, and gestational age group: **(A)** ≤27 weeks, **(B)** 28–31 weeks, **(C)** 32–36 weeks; birth years 2004–2010. Severe CP is defined as GMFCS IV-V and/or IQ < 50 (high impairment index). GMFCS, Gross motor function classification system; IQ, intellectual quotient.

The birth prevalence of CP among preterm born children varied from 7.5 per 1,000 live births in Portugal to 17.8 per 1,000 live births in Croatia. The overall prevalence and the prevalence of severe CP per birth year and GA group are given in Supplementary Table 2. Figure 1 shows time trends according to GA groups over the period considered. In children born MPT, prevalence significantly decreased linearly from 2004 to 2010 with a mean decrease of 4% per year (Incidence rate ratio (IRR) per year = 0.96 [0.92–1.00[, *p*-value 0.034). The AICs of the model indicated that a random intercept model better fitted the data compared to a random effect (random intercept and random slope) model, thus indicating that there was no significant heterogeneity between registries in the time trend. In children born VPT, the shape of time trend best fitted with a restricted cubic splines model with 3 knots, which showed a decrease until 2009 only, with no significant differences between centers. For those born EPT, we observed a steady prevalence rate over the period (IRR per year = 0.97 [0.92–1.02]). No center effect was found. Results indicated that the shape of the evolution of the prevalence of severe forms of CP (with GMFCS IV-V and/or IQ < 50) was similar to that of the overall prevalence of the corresponding GA group. The overall trend of severe CP was not significant (IRR = 0.96 [0.89–1.02]) in children born MPT, but variations in trends were observed between centers. In children born VPT, time trends of severe CP did not differ between centers, and overall no time effect was observed. In those born EPT, time effect for the prevalence of severe CP was found linear and not significant, with no differences between centers. Very similar results were observed in sensitivity analyzes whatever the cut-off of missing GA used.

The brain lesions issued from post-neonatal MRI are shown in Table 3 according to GA groups. Overall, about 50% of MRI findings were available, the availability increasing from 22 to 65% over the study period. A higher availability of MRI results was observed among CP cases with severe visual impairment (58 vs. 49% among those with no severe visual impairment) or with active epilepsy (57 vs. 47%, respectively). Predominant white matter injuries were the most common lesional patterns in all GA groups, and were more frequent in children born <32 weeks: 81.5% (≤27 weeks) and 86.4% (28–31 weeks), compared to 63.6% for children born 32–36 weeks. The proportion of children with predominant gray matter injuries increased with increasing GA, from 5.4% for those born EPT to 14.2% for those born MPT. Normal imaging related to 5.6% of children born preterm.

**Table 3:** Post neonatal brain MRI findings according to gestational age groups, 2,273 preterm born children with CP, birth years 2004 to 2010.

**MRICS *n* (%)**	**EPT ≤27 WG *n* = 499**	**VPT 28–31 WG*n* = 868**	**MPT 32–36 WG *n* = 906**
(A) Maldevelopments	7 (3.2)	7 (1.8)	33 (7.1)
(B) Predominant white matter injury	181 (81.5)	342 (86.4)	297 (63.6)
(C) Predominant gray matter injury	12 (5.4)	23 (5.8)	67 (14.3)
(D) Miscellaneous	10 (4.5)	10 (2.5)	35 (7.5)
(E) Normal	12 (5.4)	14 (3.5)	35 (7.5)
Missing	277	472	439

*EPT, extremely preterm; VPT, very preterm; MPT, moderately preterm; WG, weeks gestation; MRICS, Magnetic Resonance Imaging Classification System*.

## Discussion

### Key Findings

Our study showed that among children born preterm, only those who were born 32–36 weeks GA demonstrated a decline both in overall prevalence (significant) and prevalence of severe CP (not significant), the latter showing variations between centers. A declining trend has continued for children born 28–31 weeks between 2004 and 2009 in Europe, while children born before 28 weeks remain a matter of specific concern with a high prevalence (47.9, p. 1,000 live-births for those born in 2010), steady over the period; nearly half of them had a severe phenotype. As expected, white matter injury was the most prevalent pattern of brain lesions, with 84.6% of children born <32 weeks GA showing such brain lesions.

### Strengths and Limitations

The extended availability of GA in census data used for denominators now allows to document and compare patterns and trends of CP prevalence across GA groups from population-based CP registries. However, in this study we still could not investigate all geographical areas covered by a registry in Europe because of the unavailability of denominators by GA, thus reducing the number of children available for analyses. Besides, we must acknowledge missing values on GA but in a low proportion of children with CP, 7.6% overall. Thus, 2,273 children with CP were included in the analyses allowing precise estimates. Our analysis concentrated on the most recent period and was based on seven time points even if a long history of surveillance exists in some regions. These registries share the same definitions and classifications. Moreover, stable methodology over years limited methodological errors and provided confidence in the study findings. The rigor in data acquisition and ascertainment couldn't exclude variations in the completeness of registration between registries. Parental consent is sometimes required thereby resulting in underestimation of CP prevalence in several regions. Although completeness could have varied amongst centers, under-reporting of the most severely affected children with CP is unlikely. We, therefore, considered that changes in the prevalence of severe CP have not been affected by selection bias. All trends have to be interpreted with some caution, especially in the VPT and EPT groups because of small numbers and because points' estimates at the end of the study period have to be confirmed with additional birth years. We also observed some heterogeneity between registries in the distribution of GA in a ratio of nearly 3–1 (data not shown) that must be interpreted in line of care practices and mortality especially in EPT neonates (36) in each region. Not all registries contributed data to all the study years. We used multilevel negative binomial regression models to take into consideration such patterns and make it possible to let the average level of and temporal trend in the prevalence vary between registries. The GA-stratified analyses allowed to demonstrate specific patterns in each GA group.

### Interpretation

Our results confirmed the significant declining trend (mean linear decrease of 4% per year over the period 2004–2010) from 1983 and onwards in Europe in children born MPT or with a moderately LBW (1,500 and 2,499 g) (16, 17), consistent with reports from the Australian state of Victoria (18). Investigating a shorter period (1995–2009), Galea et al. showed a different pattern with no evidence of declining prevalence, but the prevalence rates per 1,000 LB were lower than ours for the corresponding birth years (2004–2009) except in Western Australia (20). Among children born VPT, a decrease was found until 2009. Of concern is the increase thereafter with a peak for those born in 2010, who recorded the highest rate (41.5, p. 1,000 LB) since birth year 2006. We have to note that the two centers not included in the analysis for birth years 2009–2010 (data not available, C12 and C22) showed a downward trend over the previous period 2004–2008. Therefore, higher prevalence in 2010 in the VPT group is more likely to be due to a bias linked to a reduced number of centers in the analyses rather than a true increase. However, a similar evolution was found in Western Australia (20). Both estimates were found higher to that reported in Northern Alberta, Canada (29.5 per 1,000 LB) for children with CP born VPT in 2008–2010 (25), but close to the pooled prevalence (45, p. 1,000) estimated in the meta-analysis of Pascal et al. (5). In the French population-based cohort EPIPAGE2, CP was diagnosed in 44 per 1,000 children born 27–31 weeks' gestation in 2011 (37). In this study, the median age of 24 months corrected age at diagnosis thereby should lead to an expected even higher prevalence at the age of 4–5 years, commonly considered in population-based registries.

Much attention has been given to those born before 28 weeks. In this numerically small group, reported figures and trends not only depend on the range of birth cohorts under consideration but also on the size of the populations studied. Large random variations probably affected the precision of our estimates, and some caution should be taken when interpreting the results. In the literature, various patterns of trends in CP prevalence were reported in this group. Most studies first reported an increasing prevalence until the mid-1990ies (16, 18, 38) followed by a steady (16) or downwards trend (18, 20, 38). Studies investigating the most recent period (2007 and onwards) (5, 20, 24, 25, 37, 39) mentioned highly heterogeneous prevalences from 27.2 p. 1,000 in Northern Alberta (25) to 100 p. 1,000 [meta-analysis, (5)]. In our study, the prevalence for those born EPT was in the lower range, approximatively 47–49 p. 1,000 LB from 2005 (except a peak in 2008). In this group, survival rates still highly vary especially in children born 22–23 weeks and must be taken into consideration. Thus, prevalences expressed per 1,000 neonatal survivors (NNS) are likely to be higher than prevalences per 1,000 livebirths, and should be preferred when comparing studies or periods in the most immature group of infants. In our study, the differences in the prevalences per 1,000 LB and per 1,000 NNS remained stable over the study period in regions where NNS par GA groups were available as denominators (10 centers, data not shown), with the exception of 2010 that showed a lower neonatal mortality.

A key issue was to investigate whether the most severely affected children with CP also showed a reduced prevalence thereby benefited to the same extent from optimum care for vulnerable preterm infants. No universal agreement has been reached so far to describe the severity of the condition so that substantial variation exists between studies. Information mostly focused on a combination of gross motor function loss and intellectual impairment. Incorporating various associated impairments and their severity might better reflect the overall limitations (34, 40) but possibly limits comparisons between studies. Horber and coll. reported that the proportion of those with a high impairment index did not significantly vary in children who were born PT or with a low BW between 1990 to 2006, and was very similar when considering BW or GA categories (35–39%) (34). In our study, the proportion of children with severe CP defined as non-ambulant children or children with moderate to severe intellectual disability (which corresponds to a high impairment index) slightly increased across the GA groups with 38.5% with severe phenotype among children born 32–36 weeks, up to 43.5% for those born ≤27 weeks, consistent with other studies (18, 20, 21). Our results also indicated that the prevalence of the most severely affected children with CP revealed a similar trend to the overall prevalence in the corresponding GA groups, in line with the observations previously reported by our group (16). Despite no significant time effects were found, possibly related to both the limited number of time-points and the size of sub-populations available for analyses thus reducing the chance of detecting an effect, the similarity in trends should encourage future research to focus on understanding the cumulative effect of severe associated impairments beyond to severe motor impairment.

Cerebral white matter injury is the predominant form of brain injury associated with premature birth (41). However, a large heterogeneity in the distribution of brain lesions in children born PT, and especially in the proportions of children with white matter injuries (31–71%) has been first reported in the review by Reid et al. (42). The SCPE working group has developed a MRI classification system (MRICS) for children with CP which proved highly reliable (27) and a process of regular training and feed-back is ongoing in SCPE. Using the SCPE database, Horber and coll. recently published that more than 80% of the children born <32 weeks GA had predominant white matter injury (28). Children born <32 weeks had fewer maldevelopments, a lesser proportion of gray matter injuries and consequently a greater proportion of predominant white matter injuries than children born MPT (32–36 weeks). Nagy and coll. confirmed that the distribution of brain lesions was highly correlated to perinatal data (43). As these injuries arise in the period when children are born, the decrease of prevalence in CP indicates that its major lesional cause, periventricular leukomalacia (PVL) in its severe, cystic form is decreasing. This is in line with the observation of a significant decline in the incidence of c-PVL reported in hospital based studies (44, 45), also reviewed by de Vries et al. (46). In our study, the distribution of MRI findings was comparable in children born ≤27 and 28–31 weeks, which gives hope that in the long run, also in the first group not only their mortality but also prevalence of CP will decrease.

In conclusion, in this large European study based on population-based data from CP registries, we focused on children with CP born preterm and reported a comprehensive overview of the prevalence, severity, and brain lesions according to GA groups. This paper provides evidence that a decline in prevalence of CP for those born 32–36 weeks has continued in Europe, and also for those born between 28 and 31 weeks until 2009. Trends related to the most severe phenotypes showed a similar pattern to that of overall prevalence in the corresponding GA groups.

## Data Availability Statement

The raw data supporting the conclusions of this article will be made available by the authors, without undue reservation.

## Ethics Statement

The analyses were based exclusively on pseudonymized register data compiled at European level in the SCPE database. The study did not require contact with the registered persons. Therefore, ethical review and approval were not required for this study. Each participating register has its own ethical approval that follows the legislative rules of its country. In particular procedures have been performed in accordance with the ethical standards of the institutional and/or national research committee and with the 1964 Helsinki declaration and its later amendments or comparable ethical standards. Written informed consent from the patients/ participants or patients/participants legal guardian/next of kin was not required to participate in this study in accordance with the national legislation and the institutional requirements.

## Author Contributions

CA, MD-A, DK, and IK-M designed the study. VE performed the data analysis. CA drafted the manuscript. All authors interpreted the data, contributed to revisions, and approved the final version of the manuscript.

## Conflict of Interest

The authors declare that the research was conducted in the absence of any commercial or financial relationships that could be construed as a potential conflict of interest.

## Abbreviations

BW: Birth weight
CI: confidence interval
CP: cerebral palsy
ELBW: extremely low birthweight
EPT: extremely preterm
GA: gestational age
GMFCS: Gross Motor Function Classification System
IQ: intelligence quotient
IRR: incidence rate ratio
LBW: low birthweight
MLBW: moderately low birthweight
MPT: moderately preterm
MRI: Magnetic resonance imaging
MRICS: Magnetic resonance imaging classification system
NNS: neonatal survivors
PT: Preterm
PVL: periventricular leukomalacia
SCPE: Surveillance of Cerebral Palsy in Europe
VLBW: very low birthweight
VPT: very preterm.
