# Supplementary material for: Trends in Prevalence and Severity of Pre/Perinatal Cerebral Palsy Among Children Born Preterm From 2004 to 2010: A SCPE Collaboration Study
Source: Front Neurol. 2021 May 20;12:624884. doi: 10.3389/fneur.2021.624884 (PMC8173253; doi:10.3389/fneur.2021.624884)
Supplement: Supplementary file 1 [file Table_1.docx]

Supplementary Table 1. Associated impairments by gross motor function levels according to gestational age groups, 2,273 preterm children with CP born 2004-2010

|  | | EPT  ≤27 WG  n=499  % | VPT  28-31 WG  n=868  % | MPT  32-36 WG  n=906  % |
| --- | --- | --- | --- | --- |
| GMFCS I-II  (independent walker) | Moderate to severe intellectual disability^1^  Severe visual impairment^2^  Active epilepsy^3^ | 15.1  4.1  13.6 | 5.7  2.9  8.7 | 10.4  1.6  17.1 |
| GMFCS III  (walker with aids) | Moderate to severe intellectual disability^1^  Severe visual impairment^2^  Active epilepsy^3^ | 27.6  12.7  24.1 | 17.0  4.7  15.9 | 26.1  6.7  25.8 |
| GMFCS IV-V  (wheelchair) | Moderate to severe intellectual disability^1^  Severe visual impairment^2^  Active epilepsy^3^ | 63.6  28.3  49.2 | 58.2  20.2  39.2 | 69.7  25.0  55.6 |

EPT extremely preterm ; VPT very preterm ; MPT moderately preterm ; WG weeks gestation ; GMFCS: Gross Motor Function Classification System;

^1^: intellectual quotient (IQ) <50

^2^: blind or no useful vision in both eyes

^3^: history of unprovoked seizures, still on treatment at age of registration
