# Supplementary material for: Trends in Prevalence and Severity of Pre/Perinatal Cerebral Palsy Among Children Born Preterm From 2004 to 2010: A SCPE Collaboration Study
Source: Front Neurol. 2021 May 20;12:624884. doi: 10.3389/fneur.2021.624884 (PMC8173253; doi:10.3389/fneur.2021.624884)
Supplement: Supplementary file 2 [file Table_2.docx]

Supplementary Table 2. Birth prevalence of cerebral palsy per 1,000 live births, by birth year and by gestational age group, all registries combined

|  | Overall CP prevalence per 1,000 live births  (95% confidence interval) | | | | Prevalence of severe CP per 1,000 live births  (95% confidence interval) | | | |
| --- | --- | --- | --- | --- | --- | --- | --- | --- |
| Birth year | ≤27 WG | 28-31 WG | 32-36 WG | <37 WG | ≤27 WG | 28-31 WG | 32-36 WG | <37 WG |
| 2004 | 67.0  (54.0 ; 82.1) | 47.5  (40.1 ; 55.9) | 5.3  (4.4 ; 6.3) | 12.6  (11.3 ; 13.9) | 20.7  (12.8 ; 31.4) | 14.5  (9.9 ; 20.4) | 1.9  (1.3 ; 2.7) | 4.2  (3.3 ; 5.1) |
| 2005 | 49.9  (38.8 ; 63.1) | 42.3  (35.0 ; 50.5) | 5.0  (4.1 ; 6.0) | 10.8  (9.7 ; 12.1) | 21.0  (13.2 ; 31.6) | 15.1  (10.3 ; 21.2) | 1.3  (0.8 ; 1.9) | 3.7  (2.9 ; 4.7) |
| 2006 | 47.5  (36.3 ; 61.0) | 42.2  (35.0 ; 50.4) | 5.7  (4.8 ; 6.7) | 11.0  (9.8 ; 12.2) | 23.4  (15.3 ; 34.0) | 12.8  (8.8 ; 18.1) | 2.4  (1.8 ; 3.2) | 4.3  (3.6 ; 5.2) |
| 2007 | 47.3  (36.7 ; 59.9) | 38.0  (31.6 ; 45.2) | 4.9  (4.0 ; 5.8) | 10.3  (9.2 ; 11.5) | 21.0  (13.6 ; 30.8) | 10.8  (7.3 ; 15.5) | 1.7  (1.2 ; 2.3) | 3.6  (2.9 ; 4.3) |
| 2008 | 61.0  (49.2 ; 74.5) | 35.2  (29.4 ; 41.3) | 4.5  (3.8 ; 5.2) | 9.5  (8.6 ; 10.5) | 21.9  (15.0 ; 30.8) | 11.5  (8.3 ; 15.6) | 1.2  (0.9 ; 1.7) | 3.0  (2.5 ; 3.6) |
| 2009 | 48.9  (38.1 ; 61.7) | 33.5  (27.6 ; 40.2) | 3.9  (3.3 ; 4.7) | 8.3  (7.4 ; 9.3) | 25.8  (17.2 ; 37.1) | 8.9  (5.7 ; 13.2) | 1.7  (1.2 ; 2.2) | 3.2  (2.6 ; 3.9) |
| 2010 | 47.9  (37,4 ; 60.4) | 41.5  (35.0 ; 48.8) | 4.1  (3.4 ; 4.9) | 9.4  (8.4 ; 10.5) | 15.3  (9.4 ; 23.5) | 15.8  (11.7 ; 20.7) | 1.3  (0.9 ; 1.7) | 3.2  (2.6 ; 3.8) |

CP cerebral palsy : WG weeks gestation
